# Refining the sleep circuits one neuron at a time

**DOI:** 10.1371/journal.pbio.3003101

**Published:** 2025-04-04

**Authors:** Giorgio F. Gilestro

**Affiliations:** Department of Life Sciences, Imperial College London, London, United Kingdom

## Abstract

The neural basis of sleep regulation remains elusive. A new study in PLOS Biology refines the key neuronal circuits involved in the regulation of sleep in fruit flies, confirming Drosophila melanogaster as the model of choice for unraveling the systems neuroscience of such a mysterious phenomenon.

Sleep is fundamentally a behavioral phenomenon that repeatedly suspends the conscious experience of animals for reasons we do not understand. The amount of sleep an animal requires varies dramatically across species, ranging from minutes to hours, yet in all cases, it appears governed by two key processes: a circadian drive regulating the timing for sleep and a homeostatic drive making sure that timing is obeyed. Blame homeostatic control if a sleepless night of partying makes you groggy on the following day, or if a longer-than-usual afternoon nap transforms your toddler into an energetic Godzilla at bedtime.

Courtesy of decades of work pioneered and refined in *Drosophila melanogaster*—and rightfully celebrated by the 2017 Nobel Prize—we have an excellent understanding of how the circadian clock controls the rhythmicity of sleep, at the molecular and neuronal level. Research on homeostatic control is still behind, but there are sound reasons to believe that fruit flies will be instrumental in providing crucial insights, as a study in this issue of PLoS Biology exemplifies [1].

The functional connectomics toolbox of *Drosophila* couples detailed connectivity information (the blueprint) to an arsenal of modulatory aids (the tools) to visualize or modulate neuronal firing and genetic activity. This romance between the map and the tools fuels a virtuous cycle of hypothesis generation and testing, outpacing many other models in speed, versatility, and precision.

Nowhere is this progress—and its caveats—more apparent than in the story of the dorsal fan-shaped body (dFB), a small cluster of neurons in the *Drosophila* central brain that receives input from the visual system and that was proposed a decade ago as a main regulator of sleep homeostasis. These ~30 neurons, genetically labeled by the 23E10 promoter, quickly became a focal point for sleep experiments. Early studies used the 23E10 line to silence genes, activate/inactivate neurons (via thermo- or optogenetics), and measure electrophysiological activity [2–4], leading to the tantalizing hypothesis that dFB neurons could be the first identified “somnostat”, directly controlling sleep pressure. However, this model recently faced a major challenge when two independent groups discovered that the 23E10 promoter is not exclusive to dFB neurons but also labels a cluster of neurons located in the fly’s thorax (Fig 1A). Crucially, neuronal manipulation of this previously neglected peripheral cluster alone was sufficient to alter sleep amount in a way that almost completely recapitulated the effect previously attributed to the dFB neurons in the brain [5,6].

**Fig 1:**
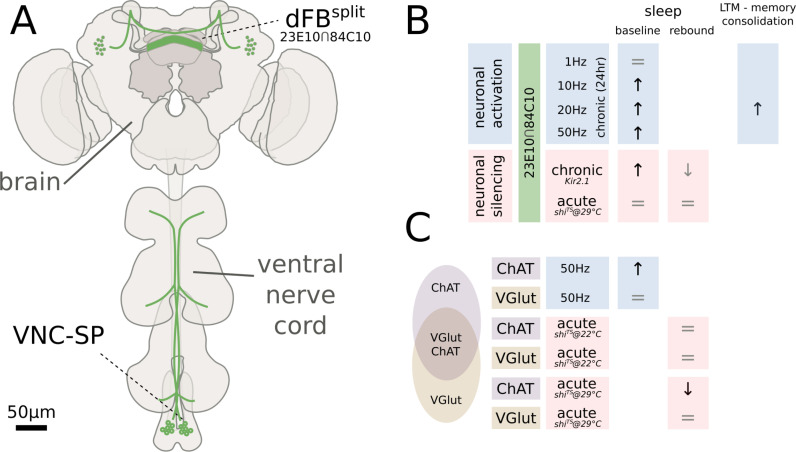
Dissecting dFB regulation of sleep in *Drosophila*. **(A)** The commonly used 23E10 GAL4 drives expression in the green cells located in the brain and the ventral nerve cord. Jones *and colleagues* have obtained a refined split-GAL4 line that limits expression in the brain cells only, excluding the peripheral VNC-SP cells. (**B)** Optogenetic stimulation of the dFBsplit cells at frequencies higher than 10 Hz for 24 h leads to an increase in baseline sleep and promotes long-term memory consolidation. Chronic inactivation also paradoxically leads to an increase in baseline sleep and mildly affects sleep homeostasis. (**C)** Optogenetics activation of cholinergic neurons also leads to an increase in baseline sleep, while inactivation leads to a decrease in sleep rebound but only in stronger regimes of thermogenetic silencing.

The finding that peripheral neurons, rather than central ones, regulate sleep was unexpected enough to forgive earlier studies that had not considered this possibility. In their latest study published in PLoS Biology, Jones *and colleagues* [1] argue, however, that a role for the dFB is not yet to be retired. Their recent, careful work suggests these neurons may still govern some aspects of sleep and even sleep-dependent memory consolidation, aligning partially with earlier studies but conflicting with some of the most recent critiques. The matter remains deliciously complicated and its suspense-rich history calls for caution. Part of the overall disagreement could be due to the fact that our understanding of what is the best way to activate a neuron is still primitive, let alone the fact that different neurons may need different experimental treatments for activation. Optogenetics and thermogenetics are fantastic tools, but the dFB literature shows that slightly different artificial firing rates (for opto-) or temperature regimes (for thermo-) can lead to radically different behavioral responses. A mild activation of the dFB neurons, for instance, seems to have no behavioral effects [1,7] or to lead to micromovements [6,8,9] when analyzed in high resolution. Jones and colleagues show [1] that a stronger and prolonged activation of the same cells, however, seems to induce something that not only looks like sleep but also has sleep’s ability to consolidate the short- to long-term memory transition (Fig 1B and 1C). Yet, so does their prolonged [1] (but not acute [1,7]) inactivation, adding to a series of interesting paradoxical observations.

The neurochemical complexity of dFB neurons further muddies the waters; they express, and often even co-express, multiple neurotransmitters and possibly peptides, forming a network that is only partly mapped. There is no consensus on whether these neurons work through—or, in fact even express—the inhibitory neurotransmitters GABA ([1] *versus* [2]) and Allatostatin A ([4] *versus* [6]), but they do seem to largely co-express Glutamate and Acetylcholine [1,10] and possibly influence sleep through a complex interaction of signals that may also require a specific neural code.

The fascinating dFB saga reflects a broader lesson: as genetic tools evolve, so must our interpretations. What began as a simple and elegant hypothesis about a sleep-control hub has unraveled into a debate about neural specificity, off-target effects, behavioral paradigms, and the interplay between central and peripheral circuits. No other animal model allows us to dissect this complex interaction quite like the fly.

## Abbreviation

dFB: fan-shaped body
